# Isotopic and Elemental Determination in Some Romanian Apple Fruit Juices

**DOI:** 10.1100/2012/878242

**Published:** 2012-04-24

**Authors:** Dana Alina Magdas, Adriana Dehelean, Romulus Puscas

**Affiliations:** Mass Spectrometry, Chromatography and Applied Physics Department, National Institute for Research and Development of Isotopic and Molecular Technologies, 65-103 Donath Street, P.O. Box 700, 400293 Cluj-Napoca 5, Romania

## Abstract

H, C, O stable isotope ratios and the content of some heavy elements of 31 Romanian single-strength organic apple juices collected from four Transylvanian areas are discussed in this study. The aim of this study was to measure the ^2^H/^1^H, ^18^O/^16^O, ^13^C/^12^C ratios of these juices and their elemental profile and to establish a database of authentic values to be used for adulteration and authenticity testing. Our results have shown mean values of *δ* ^18^O = −4.2‰ and *δ*Dδ−46.5‰, respectively, for apples from Transylvania and at the same time the mean value of *δ* ^13^C = −28.2‰. The content of Cd, Pb, U, Zn, As was below the acceptable limits stipulated in US-EPA standard for drinking water. Cu and Cr limits exceeded for one single juice; Ni content for some apple juices from Maramures, Alba, and Cluj was higher than the acceptable value.

## 1. Introduction

Fruit juices are very popular beverages, and there is an increasing demand for the required raw material all over the world. Quantity and quality of the harvested fruits are subject to significant seasonal variations depending on climatic particularities in the area of production. For example, the partial substitution of a fruit juice concentrate by the much cheaper liquid sugars (beet or cane medium invert sugar or mixture of both) or the preparation of a single-strength juice from concentrate (without proper labeling) could result in a remarkable cost advantage. Such adulteration usually does not affect consumer health; however, they are misleading and cause disadvantages for honest producers [1].

Determination of *δ* ^18^O and *δ* ^2^H values of water from fruit juices is today applied in routine analysis as an automated and acknowledged method in order to differentiate between directly pressed and rediluted single-strength juices. Authentic juices have elevated *δ* ^18^O and *δ* ^2^H content of water as compared to water from rediluted products made using tap water which is relatively depleted in heavy oxygen and hydrogen isotopes [2]. The principle of this method is an exchange of ^18^O between water and carbon dioxide equilibration with the water (juice) to be measured in a closed volume. The *δ* ^18^O values of water can be calculated from the value determinate from the carbon dioxide [2]. Also, the ^13^C/^12^C ratio measured using elemental analyzer—isotopic ratio mass spectrometry in fruit juices—has been shown to be useful for detecting the adulteration of food products [3]. Moreover, ^13^C/^12^C proved to be a good tool for characterizing geographical origin. Indeed, the *δ* ^13^C values of plant compounds are influenced by the availability of water, relative humidity, and temperature, which control stomata aperture and the internal CO_2_ concentration in the leaf [4]. In food sciences, ^13^C/^12^C ratio is a good probe for detecting the addition of cane sugar or maize glucose syrup to fruit juices [5].

The absorption of heavy metals with the diet occurs both in inorganic forms, through the corresponding salts, and as constituents of organic molecules (proteins, fats, carbohydrates, and nucleic acids). Some heavy metals (i.e., zinc, copper, iron, and selenium) are essential nutrients for health, whereas others (i.e., mercury, cadmium, lead) are toxic or have no known beneficial effects. Even the heavy metals with beneficial effects are dangerous if consumed in large quantities [6, 7]. Heavy metals may be present in foods either naturally, or by the result of human activities (manuring practices, industrial emissions, exhausted gases, etc.), or by contamination during industrial processes, preservation, and cooking [8].

Even if the concentrations of metals in beverages are normally low, a significant contribution to the increase of the metal quantity consumed by man may derive, owing to the potential great consumption of beverages. The concentration of heavy metals in fruit juices and beverages can be investigated by spectrometric technique, in both absorption and emission [9, 10] and by electroanalytical technique [11]. The introduction of inductively coupled plasma-atomic emission spectrometry and inductively coupled plasma-mass spectrometry (ICP-AES and ICP-MS) allowed a wider range of elements to be analyzed economically.

H, C, O stable isotope ratios and the content of 9 elements (Cu, Cr, Ni, Zn, Pb, Co, As, Cd, and U) of 31 Romanian apple juices collected from four Transylvanian areas are presented and discussed in this study.

## 2. Experimental

Organic apple samples were collected in the period from august 27, 2010 to September 19,2010 from different Transylvanian areas. Fruits were taken from trees, shipped in the lab, and squeezed for a week after picking them up. The variety of investigated apple was quite large; we analyzed different apple sorts like: Jonathan, Starkrimson, Golden, Melba, Legana, Classic Pippen, Stark Earliest, Voinea, and Generos of Romania, from four Transylvanian areas.

Transylvania is a Romanian province situated in the north-west towards the centre of the country, being surrounded by the Carpathians; the natural framework is made up of mountains, rivers, and plateaus. In Transylvania, the temperature can reach +35°C in summer and −25°C in winter. The average annual temperature is about 6–8°C and the average annual rainfall about 700–1000 mm/year.

### 2.1. Stable Isotope Analysis

#### 2.1.1. Sample Preparation

For oxygen-18 determination, 5 mL of raw juice (neither centrifuged nor filtered) was equilibrated with CO_2_ for 15 hours according to the CEN : ENV 13141 : 1997 method at 25 ± 0.1°C [2]. The carbon dioxide was then extracted and purified. For the hydrogen analysis, a distiller under static vacuum was used with “Rittenberg trousers” on 2-3 mL of fruit juice, always with the quantitative recovery of the water [12].

For *δ* ^13^C analysis, the separation and purification of the pulp was made according to [6, 7] by the separation of a sample of about 50 mL of fruit juice by centrifugation (10 min at 1400 times g) from the pulp. The pulp was then resuspended in water (50 mL), mixed thoroughly, and centrifuged (10 min at 1400 times g), and the supernatant was discarded. Then, the washing process was repeated twice: once with water and then with acetone; the resulting precipitate was dried under vacuum. The obtained dried solid was homogenised by mixing it with a spatula.

#### 2.1.2. Isotope Measurements

The procedure of isotope ratio mass spectrometry (IRMS) consists in measuring the isotope ratio of an analyte converted into a simple gas, isotopically representative of the original sample, before entering the ion source of an IRMS. The ^18^O isotopic of the water samples were then analyzed using a stable isotope ratio mass spectrometer IRMS (Delta V Advantage, Thermo Scientific). For *δ* ^2^H, the equipment used was a Liquid-Water Isotope Analyzer (DLT-100, Los Gatos Research). To be sure that no fractionation during the distillation process occured, we compared *δ* ^18^O results obtained with IRMS with others obtained with Liquid-Water Isotope Analyzer. The differences between the two techniques was smaller than 0.3‰. The results of our ^18^O and ^2^H analyses of the apple juices are reported using conventional *δ* notation relative to the Vienna-Standard mean Ocean Water (V-SMOW) standard (i.e., *δ*(‰) = [(*R_x_*/*R_S_*) − 1] × 1000, where *R_x_* is the ^18^O/^16^O or ^2^H/^1^H isotopic ratio of the water sample and *R_S_* is the ^18^O/^16^O or ^2^H/^1^H isotopic ratio of the V-SMOW standard.

The measurements of *δ* ^13^C from pulp fruit were carried out on an Elemental Analyser (Flash EA1112 HT, Thermo Scientific), coupled with an isotope ratio mass-spectrometer IRMS (Delta V Advantage, Thermo Scientific). For the quality control of our analysis, three working standards were analyzed at the beginning of each sequence, then three replicas from each sample were measured. NBS-22 oil with a certified value of −30.03‰  versus PDB (Pee Dee Belemnite) was used as standard. The limit of uncertainty was ±0.3‰  for *δ* ^13^C from pulp and *δ* ^18^O from juice water and ±3‰  for *δ* ^2^H values.

### 2.2. Inductively Coupled Plasma Mass Spectrometry (ICP-MS) Analysis

#### 2.2.1. Sample Preparation

The majority of ICP-MS applications involve the analysis of aqueous samples, directly or following sample pretreatment, because of the advantages of working with samples in solution. To avoid the clogging of the nebulizer, juice samples were diluted 20 times v/v.

In this survey, 2 mL of ultrapure nitric acid were added to 2 mL of apple juices in a Teflon receptacle, tightly closed. Six such receptacles were inserted in a device made of six stainless steel cylinders mounted between two flanges, to confer pressure resistance. The whole system was put in an oven at 180°C for 12 hours. A colorless solution resulted, and ultrapure water was added up to 50 mL. Thus, the apple juices samples were diluted 1 : 20 v/v.

#### 2.2.2. ICP-MS Measurements

All the determinations were carried out by the inductively coupled plasma quadrupole mass spectrometry. A Perkin Elmer ELAN DRC (e) was used with a Meinhard nebulizer and silica cyclonic spray chamber and continuous nebulization.

The operating conditions for Perkin Elmer ELAN DRC (e) were nebulizer Gas flow rates: 0.92 L/min, auxiliary Gas Flow: 1.2 L/min, plasma Gas Flow: 15 L/min, lens Voltage: 10.50 V, ICP RF Power: 1100 W, CeO/Ce = 0.023, Ba^++^/Ba^+^ = 0.021.

The operating conditions were optimized daily, by using an aqueous solution containing 10 *μ*g/L of Ba, Cd, Ce, Cu, In, Mg, Pb, Rh, U (Perkin Elmer ELAN 6100 Setup/Stab/Masscal Solution, 1% HNO_3_), and monitoring the intensities of the isotopes ^24^Mg, ^103^Rh, ^114^In, ^208^Pb, ^138^Ba, and ^140^Ce as well as the intensities at mass 69, 156, and 220 (corresponding to species ^138^Ba^2+^, ^140^Ce^16^O^+^, and background, resp.).

Calibration standards solutions and internal standards were prepared by successive dilution of a high-purity ICP-multielement calibration standard 10 *μ*g/mL of Al, As, Ba, Be, Bi, Ca, Cd, Co, Cr, Cs Cu, Fe, Ga, In, K, Li, Mg, Mn, Ni, Pb, Rb, Se, Na, Ag, Sr, Ti, V, U, Zn (Perkin Elmer Life and Analytical Sciences, Matrix: 5% HNO_3_). Ultrapure de-ionized water (18 M Ω cm^−1^) from a Milli-Q analytical reagent-grade water purification system (Millipore) and ultrapure HNO_3_ 60% (Lot-No B0157318 MERK) were used.

The relative standard deviations were better than (14.44) for Zn, (2.75) for Ni, (0.17) for Pb, (0.049) for As, (0.032) for Cd, (0.082) for Co, (0.055) for U, (34.33) for Cu, and (7.57) for Cr.

## 3. Results and Discussion

H, O stable isotope ratios from water juice and C stable isotope ratios from pulp and the content of 9 elements (Cu, Cr, Ni, Zn, Pb, Co, As, Cd, and U) of 31 Romanian apple juices collected from four Transylvanian areas are presented and discussed in this study. The aim of this study was to measure the ^2^H/^1^H, ^18^O/^16^O, ^13^C/^12^C ratios of these juices and their elemental profile and to compare these results with those already reported in literature for apple single-strength juices in order to show the particularities of Transylvanian apple juices from the 2010 harvest.

### 3.1. Isotope Ratio Mass Spectrometry (IRMS) Data

The H, O, and C stable isotope composition of plant material is generally related to the climate conditions (relative humidity, temperature, amount of precipitation), geographical characteristics (distance from the sea or other evaporation source, height, latitude) of the area where the plant grow [13–16], and the plant variety [3, 17].

By comparison with commercial fruit juices, it is known that authentic juices have elevated *δ* ^18^O and *δ* ^2^H content of water as compared to water from rediluted products made using tap water which is relatively depleted in heavy oxygen and hydrogen isotopes [2]. Even if this method of detection of illegally adulteration of commercial fruit juices is a very powerful one, it requires a sufficient number of data for authentic juices coming from different geographical origin and from different years, especially since the isotopic parameters of fruit juices show remarkable variability depending on the climate factors [1].

**Figure 1:**
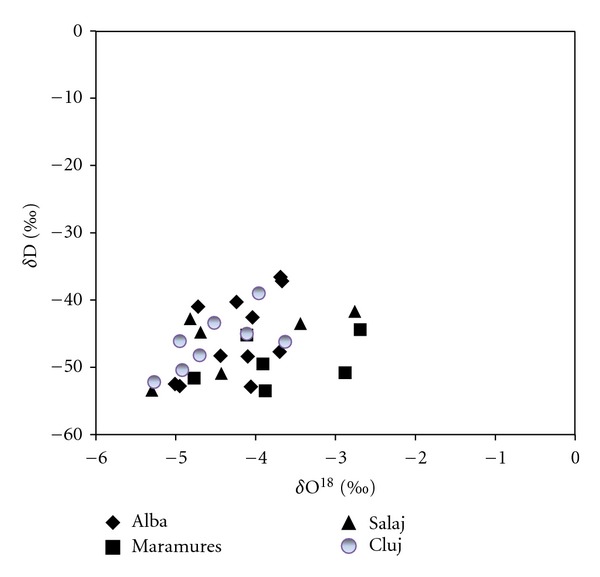
*δ* ^18^O versus *δ*D plot for investigated apple juices.

 Thus, the variation of *δ* ^18^O values in water of authentic apple single-strength juice from central Europe (Germany, Italy, Belgium, Poland, Austria, Hungary, and Czechia) for the seasons 1991–1994 obtained by Rossmann [1] showed a relatively large range of variation, between −1‰  and −5‰  with a mean value of about −4‰. The single-strength apple juices investigated by us shown a mean value of *δ* ^18^O = −4.2‰  for apples from Transylvania with small differences among the mean values for the considered regions. The variation of the mean value of each region was between −3.7‰  in Maramures region and −4.5‰  in Cluj area, and the variation range was between −2.68‰  (Maramures area) and −5.27‰  (Cluj area) (Figure 1). Determinations of *δ*D parameter of water from apple juice have shown a mean value for deuterium *δ*D *≈* −46.5‰, with a variation range of this value between −45.5‰  (Alba region) and −49‰  (Maramures region). The higher individual value that we found was −36.6‰  for Alba region, and the lower one was for Maramures region −53.5‰  (Figure 1). The lower isotopic values that we obtained for Transylvanian apple juices arise from the specific meteorological conditions of 2010 year, characterized by low temperatures and high humidity.

Photosynthetic CO_2_ assimilation via the C_3_, C_4_, and CAM pathways is of major importance in the use of carbon stable isotope ratio analysis in food authenticity control. The technique is necessarily comparative, as it must take into account the natural variation of *δ* ^13^C values in authentic products due to environmental factors such as water availability and light intensity [18].

For apple juices, Doner et al. [19] observed that the mean *δ* ^13^C value of juices from different varieties of apple and cultivated in different geographical locations was around −25.4‰, with no significant correlation between the variety of apple or geographical origin. The reported results [20] have shown that the mean *δ* ^13^C value of whole apple juice was −24.2‰  and that apples from Argentina, Mexico, and New Zeeland did not differ significantly from those grown in the USA. The *δ* ^13^C value of the fruit pulp was found to be the same as those for the corresponding fruit juice [20].

**Figure 2:**
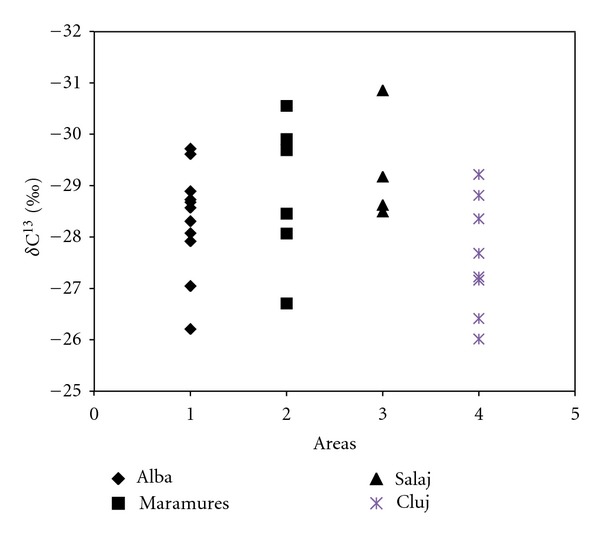
*δ* ^13^C content of apples from investigated areas.

Figure 2 presents the obtained results for *δ* ^13^C, from apple pulp, of investigated sample. The mean value *δ* ^13^C that we found was about *δ* ^13^C = −28.2‰  varying between −29.05‰  (Salaj area) and −27.6‰  (Cluj area). The higher value that we obtained for *δ* ^13^C was −26.015‰  (Cluj region), and the lower value was −30.3‰  (Salaj region). Nevertheless, the values show slight differences, due probably to environmental conditions of the plants. A possible explanation for the lowest *δ* ^13^C values obtained by us in comparison with the data reported in literature is that offered by [21], who observed in organic cabbages and onions a significantly lower *δ* ^13^C value, due to the higher microbiological activity in the soil of the organic regime resulting in respiratory CO_2_ with lower *δ* ^13^C. The results obtained by [22] in the study of organic fruits pulp revealed that factors like cultivar or growing sites resulted in more statistically significant differences in *δ* ^13^C of organic fruits than agricultural practices. In our study, no significant correlation either between the variety of apple or the geographical origin and *δ* ^13^C content was established.

### 3.2. Inductively Coupled Plasma Mass Spectrometry (ICP-MS) Data

The determination of minerals and trace metals in fruit, juice, and juice products may be performed to identify the juice authenticity, the composition of juice blends, or the geographical origin, tampering, contamination, misbranding, and adulteration of certain beverages.

The results of the present study for nine trace elements in some Romanian single-strength juice are given in Table 1.

The range of linearity of concentration versus intensity graph is of great importance in determining the elemental concentration of the juice samples. The linearity of the calibration curve was considered acceptable (the correlation factor *R* > 0.998) (Table 2).

Copper is an essential element for growth, although an emetic in large doses, but when present in beverages, certain fruit juices tend to impair the shelf life or to keep quality of such products, so it is expected that fruit juices contain relatively low levels of copper. The acute exposure to copper containing dust is manifested by metal fume fever [23].

Zinc constitutes about 33 ppm of adult body weight, and it is essential as a constituent of many enzymes involved in a number of physiological functions, such as protein synthesis and energy metabolism. Zinc deficiency, resulting from poor diet, alcoholism, and malabsorption, causes dwarfism, hypogonadism, and dermatitis, while toxicity of zinc, due to excessive intake, may lead to electrolyte imbalance, nausea, anemia, and lethargy [28]. Beside all this, both zinc and copper, two essential trace minerals, perform important biochemical functions, and they are necessary for maintaining health throughout life.

Lead and cadmium toxicity is well documented and is recognized as a major environmental health risk throughout the world. Lead affects humans and animals of all ages; however, the effects of lead are most serious in young children. Cadmium is a toxic and carcinogenic element [29, 30]. Because of their high toxicity, arsenic, lead, and cadmium need to be quantified in food and beverages [31].

The maximum acceptable limit for cadmium, lead, uranium, zinc, and copper concentration in drinking water [32] are 5 *μ*g/L, 15 *μ*g/L, 30 *μ*g/L, 5000 *μ*g/L, and 1000 *μ*g/L, respectively. In our samples, the content of Cd, Pb, U, Zn total concentration were below these limits, excepting one apple juice from Alba region which contained a higher concentration of copper (Table 1).

Nickel is an essential trace element. Human exposure to nickel may occur in industrial environment or through food chain. Nickel plays some important role in biological systems such as in enzyme activity in hormonal control and also in RNA, DNA, and protein structure or function [25]. Nickel contamination may occur during fruit processing. Upper admissible limit [32] of nickel concentration in water is 40 *μ*g/L. In our apple juice samples, this limit exceeded in Maramures, Alba, and Cluj area for some apple sorts, but the average value of Ni concentration exceeded only for Maramures area.

Ingestion in food and beverages is likely to represent the principle route of chromium intake. Chromium is a trace element, which has generated increased interest in recent years due to its essential character. Chromium acts as a cofactor in maintaining the normal metabolism of glucose [33]. Chromium upper acceptable concentration in drinking water [32] is 100 *μ*g/L. The data obtained for most samples which were analyzed are smaller that this limit, excepting one sample from Cluj area (Table 1).

In our study, the total arsenic concentration was below the acceptable limit for drinking water [32], according to US-EPA standard, for all samples.

Cobalt is a necessary cofactor for making the thyroid hormone thyroxine. Cobalt has also been used in anaemia treatment as it causes the red blood cells production. The toxicity of cobalt is quite low compared to many other metals in soil [27]. Exposure to very high levels of cobalt can cause health effects. Effects on the lungs, including asthma, pneumonia, and wheezing, have been found in workers who breathed high levels of cobalt [28].

Traces of cobalt (0.3 to 3.76 *μ*g/L for Alba area; 0.5 to 1.14 *μ*g/L for Maramures area; 0.46 to 0.94 *μ*g/L for Cluj area; 0.34 to 0.52 *μ*g/L for Salaj area) were also found.

Adraiano [34] reported lead levels of 10 *μ*g/L for beverage drink in Canada. Paolo and Maurizio [35] reported mean levels of 380 *μ*g/L lead for fruit drinks, while Contreraslopez et al. [36] reported 150 *μ*g/L lead in fruit drinks in Spain. The mean levels of lead in investigated apple fruit juice were below the levels reported by these investigators.

Paolo and Maurizio [35] and Contreraslopez et al. [36] investigated the concentrations of copper and zinc in fruit drink from Italy and Spain, respectively. They found copper in fruit drinks in a concentration range of 870–970 *μ*g/L in Italy and mean levels of 5000 *μ*g/L in Spain, while, for zinc in fruit drinks they reported 410 *μ*g/L in Italy and 5000 *μ*g/L in Spain. The levels of copper and zinc found in this study were less than mean levels reported by previous authors. 

Significant variations of elemental concentration of fruit juice among different countries [15, 24–27] were reported in literature (Table 3). Some of the results are presented in Table 3 for apple juice by comparison with the present values.

## 4. Conclusions

H, C, O stable isotope ratios and the content of 9 elements (Cu, Cr, Ni, Zn, Pb, Co, As, Cd, and U) of 31 Romanian organic apple juices collected from four Transylvanian areas are presented and discussed in this study. ICP-MS was used to analyze fruit juice samples from the point of view of heavy metals. Our data may serve as a reference for the detection of illegally adulterated apple juices. 

Our results have shown a mean value of *δ* ^18^O = −4.2‰  and *δ*D *≈* −46.5‰,  respectively, for apples from Transylvania with small differences among the apples mean values from studied regions. The variation of the mean value of each region was between −3.7‰  and −4.5‰  for *δ* ^18^O and between −45.5‰  and −49‰  for *δ*D. The mean value *δ* ^13^C that we found was about *δ* ^13^C = −28.2‰  varying between −29.05‰  (Salaj area) to −27.6‰  (Cluj area). Nevertheless, the values show slight differences, due probably to the environmental conditions of the plants. No significant correlation either between the variety of apple or the geographical origin and *δ* ^13^C content was established. 

The concentration values expressed in *μ*g/L of Ni, Zn, Cu, Cr in apple juices vary between: 10–103 *μ*g/L, 47–523 *μ*g/L, 35.6–1224 *μ*g/L, 10.6–252 *μ*g/L, respectively. Traces of Pb (0.02–11.02 *μ*g/L), Co (0.3–3.76 *μ*g/L), Cd (0.2–1.06 *μ*g/L), As (0.18–1.14 *μ*g/L), U (0.02–0.52 *μ*g/L) were also found. Our results for fruit juices were compared with the maximum limits allowed in drinking water recommended by the US-EPA also with the corresponding values of different countries available in literature. Our results have shown that the content of Cd, Pb, U, Zn, As was below the admissible limit stipulated in US-EPA standard for drinking water. Cu and Cr limits exceeded for one single juice, while Ni content was higher than the acceptable value for some apple juices from Maramures, Alba, and Cluj.

## Figures and Tables

**Table 1:** Trace elements in apple juices from Transylvania.

Element	Alba area			Maramures area			Cluj area			Salaj area		
	concentration (*μ*g/L)			concentration (*μ*g/L)			concentration (*μ*g/L)			concentration (*μ*g/L)		
	min	max	mean	min	max	mean	min	max	mean	min	max	mean
As	0.38	1.14	0.63	0.28	0.54	0.45	0.18	0.36	0.27	0.32	0.50	0.39
Cd	0.20	1.06	0.43	0.14	0.74	0.40	0.24	0.42	0.31	0.22	0.36	0.28
Pb	1.16	11.02	3.42	0.02	6.16	3.10	1.34	1.84	1.56	0.52	6.26	2.73
Co	0.30	3.76	1.22	0.50	1.14	0.66	0.46	0.94	0.64	0.34	0.52	0.49
U	0.02	0.52	0.14	0.02	0.06	0.04	0.02	0.02	0.02	0.02	0.06	0.04
Ni	10.66	51.94	22.30	16.80	103.00	45.74	16.10	81.40	34.74	12.00	24.90	17.64
Zn	95.80	523.60	281.00	89.20	432.40	178.72	56.80	72.00	65.65	76.20	133.60	99.46
Cu	136.00	1224.00	509.45	130.00	385.00	301.80	193.20	332.40	269.10	158.20	193.40	180.86
Cr	10.60	86.00	39.05	26.20	89.40	54.96	34.40	144.84	94.30	21.60	43.40	35.86

**Table 2:** Parameters of calibration curves for As, Cd, Pb, Co, U, Cu, Ni, Zn, Cr.

As	*y* = 1561.71∗*x* − 15.9921, *R* = 0.99925
Cd	*y* = 1610.89∗*x* + 2.1269, *R* = 0.999624
Pb	*y* = 9363.81∗*x* + 149.4, *R* = 0.999813
Co	*y* = 6650.49∗*x* + 11.4565, *R* = 0.999678
U	*y* = 18786.7∗*x* − 632.837, 0.998124
Cu	*y* = 3950∗*x* + 2408.12, *R* = 0.99951
Ni	*y* = 1704.03∗*x* + 1099.93, *R* = 0.999572
Zn	*y* = 1333.45∗*x* + 1190.27, *R* = 0.999526
Cr	*y* = 3536.95∗*x* + 12.6517, *R* = 0.999775

**Table 3:** Elemental composition of fruit juice among different countries versus elemental composition of Romanian apple juices.

Country	Concentration (*μ*g/L)					
	Zn	Cu	Cr	Co	Ni	Reference
France	—	—	16.00	—	—	[15]
Iran	560.00	—	—	—	—	[24]
Spain	—	—	8.00	—	—	[25]
Nigeria	474.00	535.00	10.00	—	13.00	[26]
Brazil	—	416.00	—	—	—	[26]
Brazil	—	335.70	—	—	—	[26]
Korea	—	—	—	30.00	—	[27]
Romania	156.20	315.30	56.04	0.75	30.10	Present study
